# Inflammation and salt in young adults: the African-PREDICT study

**DOI:** 10.1007/s00394-020-02292-3

**Published:** 2020-06-03

**Authors:** Simone H. Crouch, Shani Botha-Le Roux, Christian Delles, Lesley A. Graham, Aletta E. Schutte

**Affiliations:** 1 Hypertension in Africa Research Team (HART), North-West University, Potchefstroom, South Africa; 2 MRC Research Unit: Hypertension and Cardiovascular Disease, North-West University, Potchefstroom, South Africa; 3 Institute of Cardiovascular and Medical Sciences, College of Medical, Veterinary, and Life Sciences, University of Glasgow, Glasgow, UK; 4 School of Public Health and Community Medicine, University of New South Wales, The George Institute for Global Health, Sydney, Australia

**Keywords:** Sodium, Cytokine, Ethnicity, Race, African, Black

## Abstract

**Purpose:**

Low-grade inflammation and a diet high in salt are both established risk factors for cardiovascular disease. High potassium (K^+^) intake was found to counter increase in blood pressure due to high salt intake and may potentially also have protective anti-inflammatory effects. To better understand these interactions under normal physiological conditions, we investigated the relationships between 22 inflammatory mediators with 24-h urinary K^+^ in young healthy adults stratified by low, medium and high salt intake (salt tertiles). We stratified by ethnicity due to potential salt sensitivity in black populations.

**Methods:**

In 991 healthy black (*N* = 457) and white (*N* = 534) adults, aged 20–30 years, with complete data for 24-h urinary sodium and K^+^, we analysed blood samples for 22 inflammatory mediators.

**Results:**

We found no differences in inflammatory mediators between low-, mid- and high-sodium tertiles in either the black or white groups. In multivariable-adjusted regression analyses in white adults, we found only in the lowest salt tertile that K^+^ associated negatively with pro-inflammatory mediators, namely interferon gamma, interleukin (IL) -7, IL-12, IL-17A, IL-23 and tumour necrosis factor alpha (all *p* ≤ 0.046). In the black population, we found no independent associations between K^+^ and any inflammatory mediator.

**Conclusion:**

In healthy white adults, 24-h urinary K^+^ associated independently and negatively with specific pro-inflammatory mediators, but only in those with a daily salt intake less than 6.31 g, suggesting K^+^ to play a protective, anti-inflammatory role in a low-sodium environment. No similar associations were found in young healthy black adults.

**Electronic supplementary material:**

The online version of this article (10.1007/s00394-020-02292-3) contains supplementary material, which is available to authorized users.

## Introduction

Inflammation is involved in the development of cardiovascular disease [1–3]. Additionally, a diet high in salt (Na^+^) is another well-known risk factor for cardiovascular diseases, including hypertension [4]. It was recently reported that Na^+^ intake modulates the release of pro-inflammatory mediators [5–7]. These two cardiovascular risk factors may, therefore, be mechanistically involved. Interstitial Na^+^ rapidly achieves an equilibrium with plasma, and excess Na^+^ is then excreted by the kidneys [8]. However, osmotically inactive Na^+^ can also be stored in tissues, such as the skin, which in turn leads to changes in immune cell function and increased inflammation [9].

A diet high in potassium (K^+^) intake was shown to counter the usual increase in blood pressure in response to high salt intake [10, 11]. This finding suggests that a high K^+^ intake may have protective cardiovascular effects [12, 13]. As inflammation and a diet high in Na^+^ and low in K^+^ may be additive risk factors for the development of cardiovascular disease, a better understanding is required to establish the potential impact of K^+^ on cardiovascular health. As high K^+^ intake has a beneficial effect on blood pressure [14], as well as cardiovascular events and mortality [13], an additional mechanism of K^+^ may be its anti-inflammatory properties [15]. This notion is supported by a study indicating that K^+^ supplementation inhibited interleukin (IL) -17A production in human T lymphocytes that were induced by a salt load [5]. However, there is limited evidence on the role of K^+^ in the regulation of other inflammatory mediators, such as C-reactive protein (CRP), IL-6, and IL-23.

When examining Na^+^ and K^+^ handling, an essential factor to account for is black ethnicity. Black individuals have higher levels of sodium retention than their white counterparts [16]. Previous studies also reported a greater proportion of salt sensitivity in black populations [16]. The cardiovascular risk in black populations may be further increased based on their more pro-inflammatory profile when compared to white adults [17].

To better understand these potential mechanisms involved in the development of cardiovascular disease, we performed a hypothesis-generating work by investigating whether a detailed range of 22 pro- and anti-inflammatory mediators are associated with 24-h urinary K^+^ in young black and white adults. We specifically focussed on those with low, medium and high salt intake.

## Methodology

### Study population

This study forms part of the African prospective study on the early detection and identification of cardiovascular disease and hypertension (African-PREDICT) [18]. We recruited young black and white men and women, between the ages of 20 and 30 years. African-PREDICT included apparently healthy individuals who were HIV-uninfected; had a screening office brachial blood pressure of < 140 mmHg systolic and < 90 mmHg diastolic; had no self-reported previous diagnosis or used any medication for a chronic disease; and, if female, were not currently pregnant or lactating. We analysed data of participants who were included in the baseline phase of the African-PREDICT study (*n* = 1202). This study is a sub-cohort of a previously published larger cohort [17]. Participants on anti-inflammatory medication and with missing biochemical data (Na^+^, K^+^, and multiple inflammatory mediators) were additionally excluded resulting in a total of 991 participants. The exclusion of individuals with missing urine data (Na^+^ and K^+^) allowed for investigation of a more specific research question.

### Questionnaires, anthropometry and physical activity measurements

Self-reported data with regard to demographic and lifestyle information were collected using a questionnaire. A 24-h dietary recall questionnaire was administered by a trained dietitian or nutritionist on the study day and on two subsequent days. The average daily energy intake was then calculated. Socio-economic status was calculated using a point system that was adapted from Kuppuswamy’s Socio-economic Status Scale [19] for a South African environment. Height, weight and waist circumference were measured using standard methods [18]. Body mass index (BMI) was calculated using weight (kg)/height (m)^2^. A compact, chest-worn accelerometric device (Actiheart4 CamNtech Ltd and CamNtech Inc, UK) was used to objectively measure physical activity over a maximum period of 7 days.

### Ambulatory blood pressure

Participants were also fitted with a validated 24-h brachial ambulatory blood pressure monitor (Card(X)plore^®^ CE120, Meditech, Budapest, Hungary). The apparatus was programmed to record every 30 min during the day (06h00 to 22h00) and every hour during the night (22h00 to 06h00) [20]. Participants had a mean successful recording rate of 88%.

### 24-h urine collection

Participants were instructed to collect a 24-h urine sample on a day that was convenient for them and the date was noted. The first urine of the day was to be discarded and entire urine passed thereafter was collected in the provided container, including the first urine of the following morning (day 2). The start and finish time were recorded. The protocol for 24-h urine collection followed the Pan American Health Organisation/World Health Organisation (PAHO/WHO) protocol for population-level Na^+^ determination in 24-h urine samples [21]. Incomplete urine collections were defined as a volume less than 300 mL per 24 h and/or a 24-h creatinine excretion of < 4 mmol or > 25 mmol in women and < 6 mmol or > 30 mmol in men [22].

### Biological sampling and biochemical analyses

Participants fasted overnight for at least 8 h prior to attending the day of research measurements. Blood samples were collected from the median cubital vein. The samples were prepared according to the standardised protocol of the African-PREDICT study and stored at − 80 °C until analysis [18].

Urinary Na^+^, K^+^ and chloride were measured by means of ion-selective electrode potentiometry on the Cobas Integra^®^ 400 plus (Roche, Basel, Switzerland), and creatinine concentrations were measured using the Creatinine Jaffé Gen.2 reagent (Roche, Basel, Switzerland). Daily urinary Na^+^ and K^+^ excretion (mmol/day) were calculated by multiplying the Na^+^, K^+^ and creatinine concentrations (mmol/L) of the 24-h urine by the total 24-h volume of urine (in litres). Daily salt intake was estimated from the 24-h urinary Na^+^ excretion by converting Na^+^ in mmol to mg: Na^+^ (mmol) × 23 = Na^+^ (mg) [23] and then applying the conversion: 1 g salt (NaCl) = 390 mg Na^+^ [23].

A MILLIPEX Map Human High Sensitivity T-Cell Magnetic Bead Panel (EMD Millipore, Merck, MO, USA) was used to analyse 21 cytokines. This multiplex panel was analysed using Luminex xMAP technology on the Luminex 200™ analyser.

Serum samples were analysed for high-sensitivity CRP, total cholesterol, low- and high-density lipoprotein cholesterol, glucose and γ-glutamyltransferase (GGT) (Cobas Integra^®^ 400plus, Roche, Basel, Switzerland). Serum creatinine concentrations were measured using the Creatinine Jaffé Gen.2 reagent (Roche, Basel, Switzerland). Estimated creatinine clearance was determined using the Cockroft–Gault formula (Men [(140 − age) × weight in kg × 1.23]/serum creatinine or women [(140 − age) × weight in kg × 1.04]/serum creatinine). Estimated glomerular filtration rate (eGFR) was calculated using the Chronic Kidney Disease-Epidemiology (CKD-EPI) formula, without race in the equation, as the correction for race is not suggested for a South African population [24, 25]. Serum cotinine was analysed using a chemiluminescence method on the Immulite (Siemens, Erlangen, Germany) apparatus.

### Statistical analyses

IBM^®^, SPSS^®^ version 24 (IBM Corporation, Armonk, New York) was used for data analysis. GraphPad Prism 5.03 (GraphPad Software, San Diego) was used to develop all graphs. Continuous variables were inspected for normality using *Q*–*Q* plots as well as inspection of skewness and kurtosis. Variables with non-Gaussian distributions were logarithmically transformed. To substantiate the analyses by ethnicity, we investigated the interactions of ethnicity on the relationship between Na^+^, K^+^ and the full range of pro- and anti-inflammatory mediators. Based on the interactions, we divided our groups by ethnicity (Online Resource Table S1). Pro- to anti-inflammatory ratios were calculated based on the literatures [26, 27], and new ratios were suggested based on instances where pro-inflammatory mediators were higher and anti-inflammatory mediators were lower in the black and white groups. *T* test and Chi-square test were used to compare the profiles of black and white participants. We further divided our groups by Na^+^ tertiles, reflecting low, medium and high salt intake. Partial correlations and backward stepwise multiple regression were used to determine the relationship between K^+^ and pro- and anti-inflammatory mediators. Partial correlations were adjusted for age, sex and waist circumference. Variables included in backward stepwise multiple regression models were: K^+^, age, socio-economic status, AEE, waist circumference, total cholesterol, eGFR, cotinine, GGT, glucose and sex. In sensitivity analyses, we also determined whether components of the renin–angiotensin–aldosterone system contribute to the model. Multiple regression analyses displayed the last model in which potassium remained.

## Results

The general characteristics of the participants (*n* = 991) are shown in Table 1*.* The black and white groups were similar in age (24.5 years; *p* = 0.92) with an equal distribution in sex (*p* = 0.71). When viewing the detailed inflammatory mediator profile of the two groups, the black group had higher pro-to-anti-inflammatory ratios than their white counterparts (*p* ≤ 0.021) as was seen in a previous study in this population [17].

**Table 1:** Characteristics of young black and white adults

	Black (*n* = 457)	White (*n* = 534)	*p*
Age, years	24.5 ± 3.12	24.5 ± 3.04	0.94
Male, *n* (%)	227 (49.7)	259 (48.5)	0.71
Socio-economic Status			
Low, *n* (%)	264 (57.8)	109 (20.4)	**< 0.001**
Middle, *n* (%)	123 (26.9)	163 (30.5)	
High, *n* (%)	70 (15.3)	262 (49.1)	
Body composition			
Body mass index (kg/m^2^)	24.2 (17.8; 36.2)	25.0 (18.9; 35.1)	**0.014**
Waist circumference (cm)	77.6 (63.5; 98.5)	81.5 (64.9; 107)	**< 0.001**
24-h urine analysis			
Na^+^ (mmol/day)	134 (44.5; 353)	130 (45.5; 294)	0.47
Salt (NaCl g/day)	7.88 (2.62; 20.8)	7.67 (2.68; 17.3)	0.47
Above 5 g salt/day, *n* (%)	364 (79.6)	431 (80.7)	0.68
K^+^ (mmol/day)	34.5 (12.7; 98.6)	49.7 (22.3; 107)	**< 0.001**
Below 90 mmol/day K^+^, *n* (%)	441 (94.3)	460 (88.3)	**0.001**
Na^+^/K^+^	3.94 (1.93; 7.85)	2.59 (1.09; 5.37)	**< 0.001**
Inflammatory markers			
Pro-inflammatory			
CRP (mg/L)	1.02 (0.10; 12.0)	0.75 (0.08; 7.13)	**0.001**
Fractalkine (pg/mL)	28.1 (10.3; 74.4)	29.7 (10.8; 74.3)	0.15
IFN-γ (pg/mL)	6.84 (1.65; 22.0)	7.83 (1.61; 22.2)	**0.012**
IL-1β (pg/mL)	0.98 (021; 3.72)	1.10 (0.27; 3.70)	**0.031**
IL-2 (pg/mL)	0.76 (0.13; 3.88)	0.84 (0.16; 3.95)	0.16
IL-7 (pg/mL)	5.71 (1.37; 19.3)	5.63 (1.16; 18.6)	0.80
IL-8 (pg/mL)	1.75 (0.44; 6.91)	1.88 (0.47; 8.11)	0.16
IL-12 (pg/mL)	1.74 (0.36; 6.51)	1.97 (0.45; 6.72)	**0.027**
IL-17 A (pg/mL)	3.18 (0.64; 14.2)	3.53 (0.64; 14.1)	0.088
IL-23 (pg/mL)	118 (14.6; 609)	134 (12.9; 668)	0.10
ITAC (pg/mL)	4.77 (1.50; 18.0)	3.64 (1.40; 11.5)	**< 0.001**
MIP-1α (pg/mL)	9.84 (2.98; 28.4)	10.3 (2.86; 27.4)	0.34
MIP-1β (pg/mL)	7.21 (2.87; 15.7)	7.28 (2.93; 16.4)	0.76
MIP-3α (pg/mL)	2.13 (0.56; 7.68)	1.87 (0.48; 5.77)	**0.015**
TNF-α (pg/mL)	1.60 (0.42; 5.29)	1.79 (0.49; 5.76)	**0.024**
Anti-inflammatory			
IL-4 (pg/mL)	44.2 (7.97; 166)	44.6 (8.29; 154)	0.88
IL-5 (pg/mL)	0.89 (0.22; 3.90)	1.01 (0.26; 4.03)	**0.025**
IL-10 (pg/mL)	4.37 (0.94; 20.2)	5.38 (1.13; 21.2)	**< 0.001**
IL-13 (pg/mL)	3.89 (0.58; 23.3)	4.98 (0.67; 31.4)	**0.001**
Pro- and anti-inflammatory			
IL-6 (pg/mL)	1.87 (0.25; 10.3)	2.34 (0.31; 13.2)	**0.002**
IL-21 (pg/mL)	1.31 (0.21; 6.05)	1.47 (0.26; 6.47)	0.088
GM-CSF (pg/mL)	7.34 (1.19; 32.6)	8.59 (1.23; 38.0)	**0.020**
Pro-to-anti inflammatory ratios			
IL-6/IL-10	0.29 (0.04; 2.62)	0.16 (0.03; 1.22)	**< 0.001**
IL-1β/IL-10	0.22 (0.08; 0.73)	0.20 (0.07; 0.52)	**0.021**
TNF-α/IL-10	0.37 (0.16; 0.97)	0.34 (0.15; 1.01)	**0.005**
CRP/IL-10	0.23 (0.01; 4.63)	0.14 (0.01; 2.36)	**< 0.001**
MIP-1α/IL-10	2.20 (0.72; 7.04)	1.84 (0.61; 5.90)	**< 0.001**
ITAC/IL-4	0.11 (0.02; 0.82)	0.08 (0.02; 0.67)	**< 0.001**
ITAC/IL- 5	5.42 (1.16; 29.4)	3.61 (0.78; 18.1)	**< 0.001**
ITAC/IL-10	1.10 (0.29; 6.78)	0.68 (0.22; 3.05)	**< 0.001**
ITAC/IL-13	1.24 (0.18; 9.64)	0.73 (0.10; 5.90)	**< 0.001**
Biochemical markers			
Total cholesterol (mmol/L)	3.49 ± 0.98	3.98 ± 1.31	**< 0.001**
HDL-C (mmol/L)	1.14 ± 0.38	1.16 ± 0.45	0.58
LDL-C (mmol/L)	2.09 (1.01; 3.82)	2.42 (1.18; 4.39)	**< 0.001**
Triglycerides (mmol/L)	0.63 (0.31; 1.35)	0.79 (0.33; 2.05)	**< 0.001**
Glucose (mmol/L)	3.91 ± 1.05	4.23 ± 1.11	**< 0.001**
eGFR (mL/min/1.73m^2^)	123 ± 16.8	117 ± 20.3	**< 0.001**
Estimated creatinine clearance (mL/min)	138 (87.0; 235)	147 (88.7; 262)	**0.001**
Creatinine clearance (mL/min)	123 (56.4; 281)	128 (52.8; 311)	**0.28**
Plasma renin activity surrogate	63.2 (11.3; 267)	127 (38.8; 346)	**< 0.001**
Angiotensin II (pg/mL)	47.6 (8.51; 197)	94.2 (29.3; 257)	**< 0.001**
Aldosterone (pg/mL)	24.7 (5.00; 96.5)	52.3 (10.1; 223)	**< 0.001**
Ambulatory BP (mmHg)			
24 h SBP	116 ± 8.86	118 ± 9.94	**< 0.001**
24 h DBP	68.7 ± 5.73	68.5 ± 5.85	0.62
Health behaviours			
Serum cotinine (ng/mL)	3.66 (1.00; 349)	3.13 (1.00; 306)	**0.27**
Self-reported tobacco use, *n* (%)	110 (24.1)	116 (21.7)	0.37
γ-glutamyltransferase (U/L)	22.0 (8.62; 66.4)	14.7 (5.40; 48.3)	**< 0.001**
Self-reported alcohol use, *n* (%)	236 (52.4)	292 (54.8)	0.46
Hormonal contraceptive use, *n* (% of women)	105 (46.5)	112 (41.0)	0.22
Energy expenditure			
TEE (kcal/day)	2218 ± 394	2355 ± 497	**< 0.001**
AEE (kcal/day)	430 ± 219	406 ± 204	0.12
Reported energy intake			
Energy intake (kcal/day)	2097 ± 806	2083 ± 710	0.82

Bold values indicate *p* < 0.05. Data presented as mean ± SD; or geometric mean 95 CI. Granulocyte–macrophage colony-stimulating factor (GM-CSF), interferon gamma (IFN-γ), interleukin 1 beta (IL-1β), interleukin 2 (IL-2), Interleukin 4 (IL-4), interleukin 5 (IL-5), interleukin 6 (IL-6), interleukin 7 (IL-7), interleukin 8 (IL-8), interleukin 10 (IL-10), interleukin 12 (IL-12), interleukin 13 (IL-13), interleukin 17A (IL-17A), interleukin 21 (IL-21), interleukin 23 (IL-23), interferon-inducible T-cell alpha chemoattractant (ITAC), macrophage inflammatory protein 1-alpha (MIP-1α), macrophage inflammatory protein 1-beta (MIP-1β), Macrophage inflammatory protein 3-alpha (MIP-3α) and tumour necrosis factor alpha (TNFα), SBP, systolic blood pressure; DBP, diastolic blood pressure; BMI, body mass index; HDL-C high density lipoprotein cholesterol; LDL-C, low density lipoprotein chole sterol

There were no ethnic differences for Na^+^ excretion (*p* = 0.47), but black participants had lower urine levels of K^+^, with 94% black and 88% white participants having K^+^ levels below recommended levels [28]. Black participants had higher Na^+^/K^+^ ratios (*p* < 0.001) than the white group.

We determined the differences in inflammatory mediator concentrations according to Na^+^ tertiles (Online Resource Table S2). For all inflammatory mediators, there were generally no differences.

To establish whether a relationship exists between Na^+^ or K^+^ with inflammatory mediators, we performed partially adjusted regression analyses in the total group as well as black and white groups separately (adjusted for age, sex and waist circumference as well as ethnicity in the total group) (Online Resource Table S3). These analyses yielded minimal correlations mostly with K^+^ as indicated in detail in Online Resource Table S3*.*

**Fig. 1:**
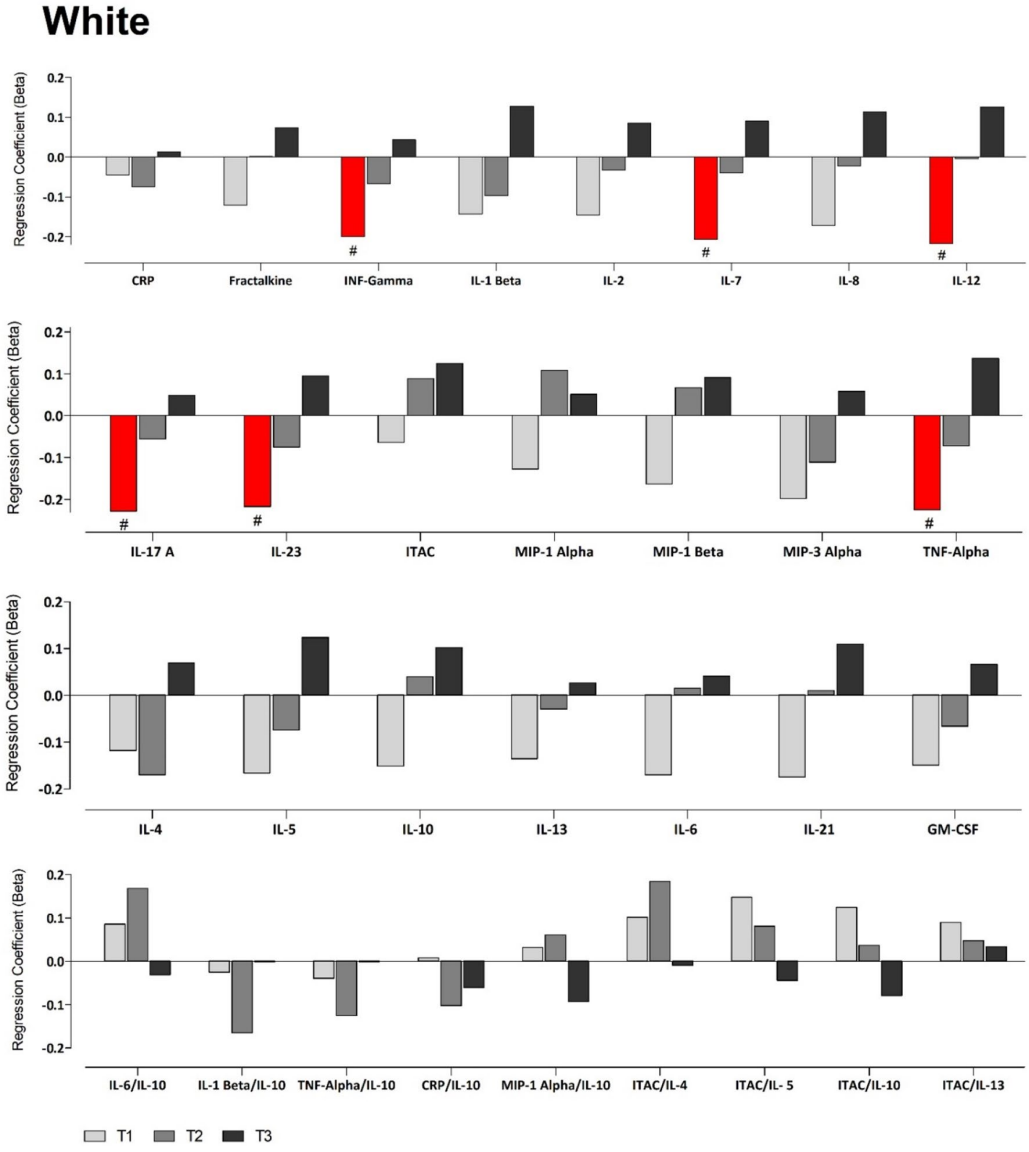
Multi-variable adjusted regression analyses showing the relationship between inflammatory mediators and K^+^ within each Na^+^ tertile in white adults. Each model was adjusted for: age, sex, socio-economic status, waist circumference, total cholesterol, glucose, gamma glutamyltransferase, cotinine, estimated glomerular filtration rate, activity energy expenditure. ^#^*p* < 0.05

Due to previous reports indicating the importance of Na^+^/K^+^ balance [29], we then performed partial correlations between K^+^ and inflammatory mediators in the groups stratified by Na^+^ tertiles. In whites, we found several prominent results in the lowest Na^+^ tertile (T1). These include positive correlations between K^+^ and both interferon-inducible T-cell alpha chemoattractant (ITAC)/IL-5 and ITAC/IL-10. In T1, we also found negative correlations between K^+^ and interferon gamma (IFN-γ), IL-1β, IL-5, IL-6, IL-7, IL-8, IL-12, IL-17A, IL-21, IL-23, macrophage inflammatory protein 3-alpha (MIP-3α) and tumour necrosis factor alpha (TNF-α). Additionally, in the middle tertile (T2), K^+^ correlated inversely with IL-4 (Online Resource Fig. S1). In fully adjusted regression analyses (Fig. 1), these findings were confirmed where K^+^ associated negatively with the pro-inflammatory mediators IFN-γ, IL-7, IL-12, IL-17A, IL-23 and TNF-α, but only in the lowest Na^+^ tertile T1 (all *p* ≤ 0.046).

**Fig. 2:**
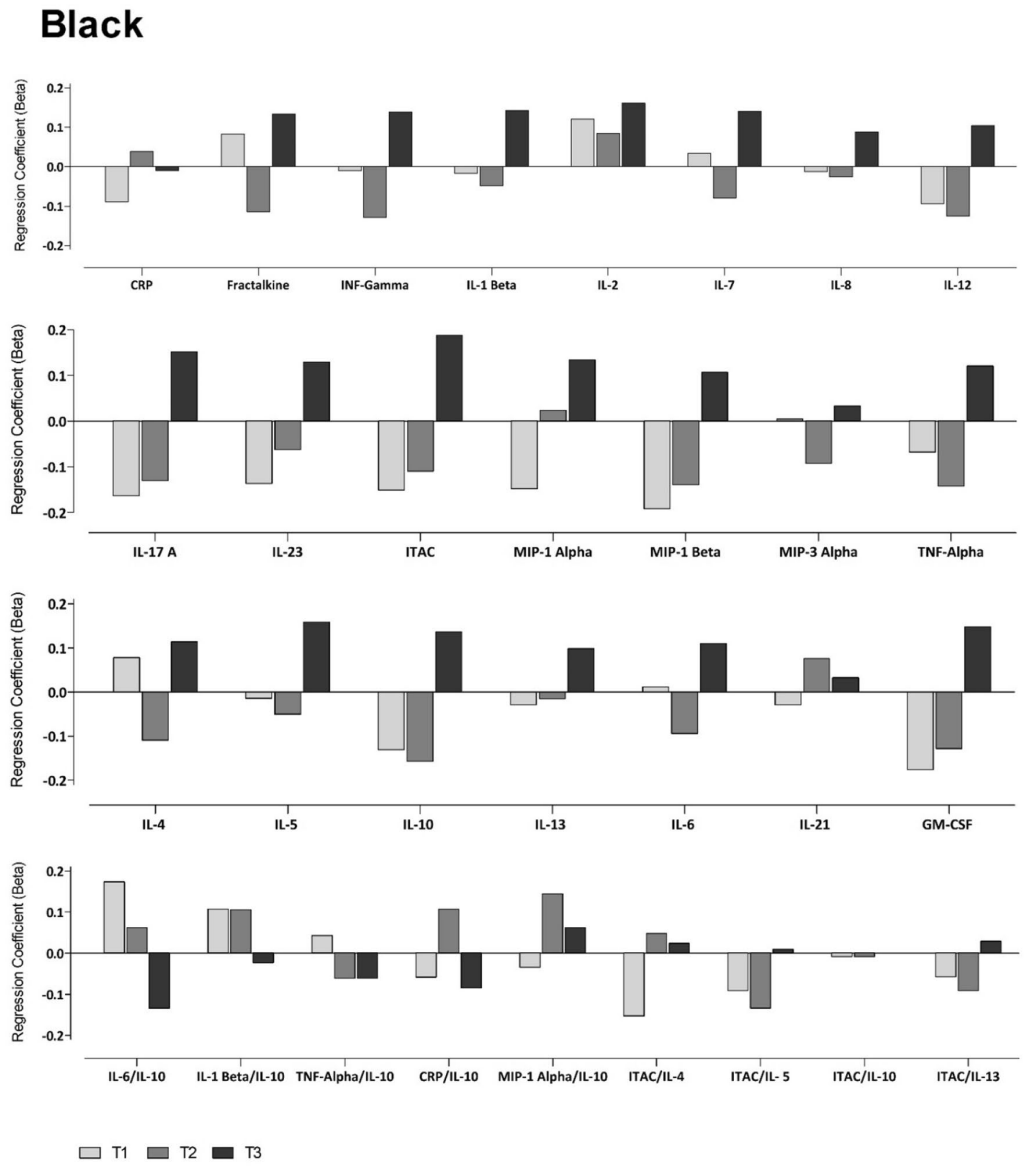
Multi-variable adjusted regression analyses showing the relationship between inflammatory mediators and K^+^ within each Na^+^ tertile in black adults. Each model was adjusted for: age sex, socio-economic status, waist circumference, total cholesterol, glucose, gamma glutamyltransferase, cotinine, estimated glomerular filtration rate, activity energy expenditure. ^#^*p* < 0.05

In the black population, with partial correlations, we found in the highest Na^+^ tertile (T3) positive correlations between K^+^ and both ITAC and IL-5, and negative correlations in the lowest tertile (T1) with ITAC/IL-4 and ITAC/IL-5 (all *p* ≤ 0.046) (Online Resource Fig. S2). However, these results lost significance in fully adjusted regression analyses (Fig. 2). We examined renin, angiotensin II and aldosterone’s impact on the model, all of which exhibited no effect (results not shown). We additionally examined the relationship between Na^+^ and inflammatory mediators, stratified by Na^+^, but found no significant correlations.

## Discussion

Low-grade systemic inflammation and Na^+^ are both risk factors for the development of cardiovascular disease [1–4]. It has been suggested that K^+^ may provide a protective anti-inflammatory effect [15]. Therefore, to better understand the possible mechanisms through which a high-salt environment may predispose one to higher cardiovascular disease risk (potentially due to the loss of the ‘protective’ anti-inflammatory role of K^+^), we examined the relationships between a detailed range of inflammatory mediators and 24-h urinary K^+^, in those with low, medium and high salt intake. When we stratified 991 young healthy black and white participants by Na^+^ excretion tertiles, we found negative independent relationships between urinary K^+^ and six pro-inflammatory mediators IFN-γ, IL-7, IL-12, IL-17A, IL-23 and TNF-α, but only in white adults and only in those within the lowest Na^+^ tertile (with an equivalent of 4.21 [0.63–6.31] g salt intake/day). These findings suggest that K^+^ may exert protective anti-inflammatory functions, but only in individuals with a low salt intake as reflected by the 24-h urinary Na^+^ excretion.

Previous studies have shown that a diet high in Na^+^ stimulates an inflammatory response [6, 8, 30]. In healthy human participants participating in the Mars520 study, Titze et al*.* found an increase in the pro-inflammatory mediators IL-6 and IL-23, as well as a decrease in the anti-inflammatory mediator IL-10 in those on a high-salt diet [7].

In support of our findings of several negative relationships between pro-inflammatory mediators and urinary K^+^, it was found that rats on a K^+^-supplemented diet had suppressed renal inflammation [15]. This was evident by a decrease in macrophage infiltration and nuclear factor kappa B (NF-κB), as well as a lower expression of cytokines [15]. In addition, a study involving healthy humans found K^+^ supplementation to have an inhibiting effect on the production of IL-17A by T lymphocytes induced by salt loading [5]. One potential mechanism through which K^+^ may suppress inflammation is via its anti-oxidant effect [5]. Increase in extracellular K^+^ leads to elevated membrane-Na^+^ pump activity [31]. This in turn results in hyperpolarization and ultimately a reduction in oxidase activity [31]. A second proposed mechanism is via K^+^ inhibiting the effects of Na^+^ on mitogen-activated protein kinase p38 which, when activated, leads to an immune response [5]. It has also been suggested that K^+^ may suppress the activation of NF-κB, which is involved in regulating genes relating to inflammation in the kidneys [15, 32, 33].

When we examined the relationship between K^+^ and inflammation, inverse relationships were seen with pro-inflammatory mediators, but not with anti-inflammatory mediators. This suggests a potential role of K^+^ in pro-inflammatory processes. What is of particular interest is that this protective association is only seen in the lowest Na^+^ tertile, with an average salt intake of 4.21 (0.63–6.31) g/day (or 10.7–107 mmol Na^+^/day). The mean intake for the second and third Na^+^ tertiles in the white group were 8.13 (6.31–10.0) g salt/day and 13.9 (10.0–50.1) g salt/day, respectively. These findings suggest that once Na^+^ intake exceeds the levels of the first Na^+^ tertile, or when the Na^+^/K^+^ equilibrium becomes significantly imbalanced, the protective effect of K^+^ may be lost. This may imply that while it is important to maintain an acceptable Na^+^/K^+^ ratio, it is also of importance to do so at the recommended levels. Our findings, thus, suggest a loss of mediation of pro-inflammatory mediators by K^+^ in individuals with increased Na^+^ intake.

As previously mentioned, it is also important to consider the role of ethnicity on the relationship between inflammatory mediators, K^+^ and Na^+^. While numerous studies have examined differences in inflammation between ethnic groups, global findings remain contradictory [34]. However, multiple studies performed in South African populations have found that black individuals display higher levels of pro-inflammatory markers and an overall more pro-inflammatory profile [17, 35–37]. When examining Na^+^, previous studies found that black adults have a predisposition for higher Na^+^ retention [16]. Based on previous reports, looking at salt sensitivity, black populations also have a greater response in blood pressure to Na^+^ [38]. Regardless, research into the role of Na^+^ and K^+^ in inflammation in any populations, but particularly black populations, is limited. While some studies have, to a limited extent, examined the role of K^+^ in inflammation [5, 15], to the best of our knowledge, none have examined this relationship stratified by ethnicity. This is of importance as studies have found ethnic differences in K^+^ excretion, with black populations being found to excrete less K^+^ than their white counterparts even when intake is matched [39].

Our findings were only present in the white group. Although, a previous study found that K^+^ supplementation protects against an increase in blood pressure in black populations in response to a salt load [10]. In our study with the focus on inflammation, this potentially protective effect on blood pressure was not seen in terms of potential anti-inflammatory effects. It is unknown whether this lack of association in the black group may be due to the effects of salt sensitivity. It should, however, be taken into account that the black group had particularly low urinary K^+^ levels. Only 6% of the black population had a K^+^ intake above the recommended minimum of 90 mmol/day [28], which may be a reason for the lack of association in this group. While protective associations are seen in the white adults, their mean K^+^ intake was also below the recommended daily K^+^ intake, albeit to a lesser extent than the black population. It would certainly be worth investigating whether an increase in K^+^ intake in both groups would result in greater anti-inflammatory responses. However, it is important to note that an increase in K^+^ levels should not be achieved by increasing calorie intake, but rather through the consumption of foods high in K^+^, such as fruits and vegetables [40].

A strength of our study is the absence of pre-existing chronic diseases, which gave us the opportunity to test our hypotheses in adults without an influence from pathology. Additionally, our study included a large panel of pro- and anti-inflammatory mediators which were analysed with a high-sensitivity kit. Although we included the renin–angiotensin–aldosterone system components in regression models, which yielded no contributory findings, the renin–angiotensin–aldosterone system is likely to be very important perhaps in those who have developed hypertension. In terms of limitations, the use of a single collection of 24‐h urine does not account for day‐to‐day variations in Na^+^ and K^+^ excretion.

In conclusion, in young apparently healthy white adults, we found significant negative relationships between 24-h urinary K^+^ and specific pro-inflammatory mediators, but only in those with a daily salt intake of less than 6.31 g. Our results suggest that K^+^ may play a protective, anti-inflammatory role in a low-sodium environment.

## Electronic supplementary material

Below is the link to the electronic supplementary material.

Supplementary file1 (DOCX 14 kb)

Supplementary file2 (DOCX 29 kb)

Supplementary file3 (DOCX 22 kb)

Supplementary file4 (DOCX 214 kb)

Supplementary file5 (DOCX 201 kb)

## Data Availability

The study methodology has been published [18], whereas the data dictionary, statistical analysis, protocol and deidentified individual participant data will be made available upon reasonable request to the corresponding author in agreement with all co-authors.

## Abbreviations

CRP: C-reactive protein
Na^+^: Sodium
K^+^: Potassium
GM-CSF: Granulocyte–macrophage colony-stimulating factor
IFN-γ: Interferon gamma
IL-1 β: Interleukin 1 beta
IL-2: Interleukin 2
IL-4: Interleukin 4
IL-5: Interleukin 5
IL-6: Interleukin 6
IL-7: Interleukin 7
IL-8: Interleukin 8
IL-10: Interleukin 10
IL-12: Interleukin 12
IL-13: Interleukin 13
IL-17A: Interleukin 17A
IL-21: Interleukin 21
IL-23: Interleukin 23
ITAC: Interferon-inducible T-cell alpha chemoattractant
MIP-1α: Macrophage inflammatory protein 1-alpha
MIP-1β: Macrophage inflammatory protein 1-beta
MIP-3α: Macrophage inflammatory protein 3-alpha
NF-κB: Nuclear factor kappa B
TNFα: Tumour necrosis factor alpha
