# Supplementary material for: Inflammation and salt in young adults: the African-PREDICT study
Source: Eur J Nutr. 2020 Jun 3;60(2):873–82. doi: 10.1007/s00394-020-02292-3 (PMC7900065; doi:10.1007/s00394-020-02292-3)
Supplement: Supplementary file 1 — Supplementary file1 (DOCX 14 kb) [file 394_2020_2292_MOESM1_ESM.docx]

**Inflammation and salt in young adults:**

**the African-PREDICT study**

**European Journal of Nutrition**

Simone H Crouch,^a^ Shani Botha-Le Roux,^a,b^ Christian Delles,^c^ Lesley A Graham,^c^ Aletta E Schutte^a,b,d^

*^a^ Hypertension in Africa Research Team (HART), North-West University, Potchefstroom, South Africa*

*^b^ MRC Research Unit: Hypertension and Cardiovascular Disease, North-West University, Potchefstroom, South Africa*

*^c^ Institute of Cardiovascular and Medical Sciences, College of Medical, Veterinary, and Life Sciences, University of Glasgow, Glasgow, United Kingdom*

*^d^ School of Public Health and Community Medicine, University of New South Wales; The George Institute for Global Health, Sydney, Australia*

Corresponding author: Prof. AE Schutte, School of Public Health and Community Medicine, Faculty of Medicine, Kensington Campus, High Street, Randwick, Sydney 2052, Email: a.schutte@unsw.edu.au

| **Table S1.** Interactions of ethnicity on cytokines | | |
| --- | --- | --- |
|  | **Na^+^** | **K^+^** |
|  | *p* | *p* |
| Fractalkine | 0.43 | 0.29 |
| CRP | 0.71 | 0.16 |
| GM-CSF | 0.55 | 0.29 |
| IFN *gamma* | 0.55 | 0.15 |
| IL-1 *beta* | 0.90 | 0.16 |
| IL-2 | 0.15 | **0.022** |
| IL-4 | 0.36 | 0.26 |
| IL-5 | 0.37 | **0.019** |
| IL-6 | 0.98 | 0.16 |
| IL-7 | 0.89 | 0.15 |
| IL-8 | 0.96 | 0.40 |
| IL-10 | 0.95 | 0.35 |
| IL-12 | 0.76 | 0.25 |
| IL-13 | 0.81 | 0.11 |
| IL-17 A | 0.31 | 0.20 |
| IL-21 | 0.56 | 0.18 |
| IL-23 | 0.61 | 0.34 |
| ITAC | 0.46 | 0.77 |
| MIP *1*-*alpha* | 0.31 | 0.54 |
| MIP *1*-*beta* | 0.77 | 0.84 |
| MIP 3-*alpha* | 0.58 | 0.62 |
| TNF *alpha* | 0.98 | 0.28 |
