# Supplementary material for: Inflammation and salt in young adults: the African-PREDICT study
Source: Eur J Nutr. 2020 Jun 3;60(2):873–82. doi: 10.1007/s00394-020-02292-3 (PMC7900065; doi:10.1007/s00394-020-02292-3)
Supplement: Supplementary file 2 — Supplementary file2 (DOCX 29 kb) [file 394_2020_2292_MOESM2_ESM.docx]

**Inflammation and salt in young adults:**

**the African-PREDICT study**

**European Journal of Nutrition**

Simone H Crouch,^a^ Shani Botha-Le Roux,^a,b^ Christian Delles,^c^ Lesley A Graham,^c^ Aletta E Schutte^a,b,d^

*^a^ Hypertension in Africa Research Team (HART), North-West University, Potchefstroom, South Africa*

*^b^ MRC Research Unit: Hypertension and Cardiovascular Disease, North-West University, Potchefstroom, South Africa*

*^c^ Institute of Cardiovascular and Medical Sciences, College of Medical, Veterinary, and Life Sciences, University of Glasgow, Glasgow, United Kingdom*

*^d^ School of Public Health and Community Medicine, University of New South Wales; The George Institute for Global Health, Sydney, Australia*

Corresponding author: Prof. AE Schutte, School of Public Health and Community Medicine, Faculty of Medicine, Kensington Campus, High Street, Randwick, Sydney 2052, Email: a.schutte@unsw.edu.au

| **Table S2. Analysis of variance between Na^+^ tertiles T1, T2 and T3 in black and white adults.** | | | | | | | | | | | | | | | | | | | | | | | | | | | | | | | |
| --- | --- | --- | --- | --- | --- | --- | --- | --- | --- | --- | --- | --- | --- | --- | --- | --- | --- | --- | --- | --- | --- | --- | --- | --- | --- | --- | --- | --- | --- | --- | --- |
|  | **Black** | | | | | | | |  | | | | | | **White** | | | | | | | | | | | | | | | |  |
|  | **T1** | | | | **T2** | | | **T3** | | | **p** | | | | **T1** | | | | | | **T2** | | | | | **T3** | | | | **p** | |
| **24hr Urine Analysis** |  | | |  | | | |  | | | |  | | | |  | | |  | | | | |  | | | | |  | | |
| Na^+^ (mmol/day) | 67.5^ab^ (63.0; 72.2) | | | | 134^ac^ (131; 137) | | | 248^bc^ (237; 259) | | | **<0.001** | | | | 71.4^ab^ (67.7; 75.3) | | | | | 138^ac^ (135; 140) | | | | | 236^bc^ (226; 247) | | | | **<0.001** | | |
| Salt (NaCl g/day) | 3.98^ab^ (3.71; 4.26) | | | | 7.92^ac^ (7.74; 8.10) | | | 14.6^bc^ (14.0; 15.3) | | | **<0.001** | | | | 4.21^ab^ (3.99; 4.44) | | | | | 8.13^ac^ (7.97; 8.29) | | | | | 13.9^bc^ (13.3; 14.6) | | | | **<0.001** | | |
| K^+^ (mmol/day) | 21.1^ab^ (19.4; 23.0) | | | | 33.5^ac^ (31.4; 35.7) | | | 53.6^bc^ (49.6; 58.0) | | | **<0.001** | | | | 36.4^ab^ (33.9; 39.1) | | | | | 50.1^ac^ (46.6; 53.9) | | | | | 69.2^bc^ (65.3; 73.4) | | | | **<0.001** | | |
| Na^+^/K^+^ | 3.23^ab^ (2.97; 3.52) | | | | 4.03^ac^ (3.79; 4.28) | | | 4.58^bc^ (4.29; 4.89) | | | **<0.001** | | | | 1.97^ab^ (1.82; 2.14) | | | | | 2.66^ac^ (2.50; 2.83) | | | | | 3.41^bc^ (3.23; 3.59) | | | | **<0.001** | | |
|  |  | | | |  | | |  | | |  | | | |  | | | | |  | | | | |  | | | |  | | |
| **Biochemical Analyses** | | | | |  | | |  | | |  | | | |  | | | | |  | | | | |  | | | |  | | |
| Creatinine Clearance  (mL/min) | 82.5^ab^ (76.7; 88.8)) | | | | 117^ac^ (109; 125) | | | 175^bc^ (164; 187) | | | **<0.001** | | | | 91.8^ab^ (84.9; 99.2) | | | | | 134^ac^ (126; 143) | | | | | 175^bc^ (163;189) | | | | **<0.001** | | |
| Plasma renin activity surrogate | 72.8^a^ (62.4; 84.9) | | | | 62.4 (53.8; 72.5) | | | 54.7^a^ (47.1; 63.5) | | | **0.029** | | | | 145^a^ (131; 160) | | | | | 125 (114; 137) | | | | | 110^a^ (99.0; 121) | | | | **<0.001** | | |
| Angiotensin II (pg/mL) | 54.6^a^ (46.8; 63.8) | | | | 47.2 (40.5; 54.9) | | | 41.2^a^ (35.2; 48.1) | | | **0.036** | | | | 105^a^ (95.2; 116) | | | | | 93.3 (84.5; 103) | | | | | 83.9^a^ (75.7; 92.9) | | | | **0.008** | | |
| Aldosterone (pg/mL) | 30.2^ab^ (26.3; 34.6) | | | | 22.2^a^ (19.4; 25.4) | | | 22.2^b^ (19.7; 25.2) | | | **0.001** | | | | 63.9^a^ (56.0; 73.0) | | | | | 51.9 (45.0; 59.9) | | | | | 43.2^a^ (38.0; 49.1) | | | | **<0.001** | | |
|  |  | | | |  | | |  | | |  | | | |  | | | | |  | | | | |  | | | |  | | |
| **Inflammatory Markers** | | | | |  | | |  | | |  | | | |  | | | | |  | | | | |  | | | |  | | |
| *Pro-Inflammatory* |  | | | |  | | |  | | |  | | | |  | | | | |  | | | | |  | | | |  | | |
| CRP (mg/L) | 1.05 (0.81; 1.36) | | | | 1.02 (0.78; 1.32) | | | 1.01 (0.80; 1.26) | | | 0.97 | | | | 0.82 (0.65; 1.03) | | | | | 0.71 (0.59; 0.86) | | | | | 0.72 (0.59; 0.88) | | | | 0.56 | | |
| Fractalkine (pg/mL) | 27.6 (25.0; 30.5) | | | | 28.7 (25.7; 32.0) | | | 28.0 (25.4; 30.8) | | | 0.87 | | | | 29.8 (27.1; 32.8) | | | | | 28.3 (25.9; 30.9) | | | | | 31.3 (28.4; 34.5) | | | | 0.32 | | |
| IFN-γ (pg/mL) | 6.92 (6.07; 7.88) | | | | 7.30 (6.37; 8.37) | | | 6.41 (5.63; 7.29) | | | 0.38 | | | | 7.94 (7.04; 8.95) | | | | | 7.53 (6.63; 8.54) | | | | | 8.05 (7.10; 9.13) | | | | 0.73 | | |
| IL-1β (pg/mL) | 0.92 (0.80; 1.06) | | | | 1.02 (0.89; 1.16) | | | 1.00 (0.86; 1.16) | | | 0.59 | | | | 1.10 (0.97; 1.24) | | | | | 1.05 (0.94; 1.18) | | | | | 1.15 (1.03; 1.29) | | | | 0.56 | | |
| IL-2 (pg/mL) | 0.70 (0.59; 0.84) | | | | 0.79 (0.65; 0.95) | | | 0.79 (0.65;0.96) | | | 0.62 | | | | 0.86 (0.74; 0.99) | | | | | 0.82 (0.70; 0.94) | | | | | 0.83 (0.70; 0.98) | | | | 0.89 | | |
| IL-7 (pg/mL) | 5.57 (4.93; 6.30) | | | | 5.85 (5.08; 6.74) | | | 5.71 (5.03; 6.48) | | | 0.88 | | | | 5.47 (4.79; 6.24) | | | | | 5.32 (4.70; 6.03) | | | | | 6.21 (5.48; 7.03) | | | | 0.21 | | |
| IL-8 (pg/mL) | 1.77 (1.54; 2.05) | | | | 1.71 (1.50; 1.96) | | | 1.75 (1.53; 2.00) | | | 0.94 | | | | 1.81 (1.61; 2.05) | | | | | 1.93 (1.70; 2.19) | | | | | 1.91 (1.69; 2.14) | | | | 0.75 | | |
| IL-12 (pg/mL) | 1.76 (1.53; 2.02) | | | | 1.76 (1.50; 2.04) | | | 1.71 (1.53; 1.97) | | | 0.95 | | | | 1.97 (1.73; 2.24) | | | | | 1.86 (1.62; 2.14) | | | | | 2.10 (1.87; 2.37) | | | | 0.43 | | |
| IL-17 A (pg/mL) | 3.04 (2.60; 3.55) | | | | 3.37 (2.84; 3.95) | | | 3.17 (2.74; 3.67) | | | 0.66 | | | | 3.53 (3.07; 4.07) | | | | | 3.45 (3.00; 3.97) | | | | | 3.63 (3.14; 4.20) | | | | 0.88 | | |
| IL-23 (pg/mL) | 121 (102; 144) | | | | 121 (99.5; 148) | | | 113 (93.2; 136) | | | 0.82 | | | | 132 (109; 158) | | | | | 131 (109; 157) | | | | | 140 (116; 167) | | | | 0.87 | | |
| ITAC (pg/mL) | 4.85 (4.27; 5.50) | | | | 4.86 (4.23; 5.59) | | | 4.62 (4.10; 5.20) | | | 0.81 | | | | 3.46 (3.13; 3.81) | | | | | 3.79 (3.40; 4.22) | | | | | 3.70 (3.35; 4.10) | | | | 0.42 | | |
| MIP-1α (pg/mL) | 9.66 (8.61; 10.8) | | | | 10.1 (8.97; 11.4) | | | 9.78 (6.68; 11.0) | | | 0.86 | | | | 9.72 (8.70; 10.9) | | | | | 10.8 (9.74; 12.0) | | | | | 10.4 (9.26; 11.7) | | | | 0.39 | | |
| MIP-1β (pg/mL) | 7.25 (6.68; 7.87) | | | | 7.36 (6.75; 8.03) | | | 7.04 (6.44;7.69) | | | 0.75 | | | | 7.03 (6.48; 7.63) | | | | | 7.33 (6.75; 7.69) | | | | | 7.51 (6.89; 8.19) | | | | 0.54 | | |
| MIP-3α (pg/mL) | 2.02 (1.76; 2.31) | | | | 2.32 (2.03; 2.65) | | | 2.08 (1.85; 2.36) | | | 0.31 | | | | 1.70 (1.48; 1.95) | | | | | 1.94 (1.74; 2.17) | | | | | 1.99 (1.75; 2.27) | | | | 0.17 | | |
| TNF-α (pg/mL) | 1.57 (1.38; 1.78) | | | | 1.65 (1.45; 1.87) | | | 1.60 (1.41; 1.82) | | | 0.87 | | | | 1.70 (1.51; 1.91) | | | | | 1.76 (1.57; 1.98) | | | | | 1.94 (1.74; 2.17) | | | | 0.25 | | |
| *Anti-Inflammatory* |  | | | |  | | |  | | |  | | | |  | | | | |  | | | | |  | | | |  | | |
| IL-4 (pg/mL) | 46.2 (40.3; 52.9) | | | | 44.3 (37.6; 52.2) | | | 42.4 (36.5; 49.2) | | | 0.72 | | | | 46.5 (40.7; 53.4) | | | | | 39.8 (34.4; 46.1) | | | | | 48.4 (42.3; 55.4) | | | | 0.11 | | |
| IL-5 (pg/mL) | 0.88 (0.76; 1.01) | | | | 0.88 (0.74; 1.04) | | | 0.91 (0.78; 1.05) | | | 0.95 | | | | 1.02 (0.90; 1.16) | | | | | 0.99 (0.87; 1.13) | | | | | 1.02 (0.90; 1.16) | | | | 0.92 | | |
| IL-10 (pg/mL) | 4.40 (3.81; 5.09) | | | | 4.56 (3.89; 5.35) | | | 4.18 (3.58; 4.88) | | | 0.72 | | | | 5.39 (4.69; 6.19) | | | | | 5.36 (4.70; 6.11) | | | | | 5.41 (4.73; 6.20) | | | | 0.99 | | |
| IL-13 (pg/mL) | 3.69 (3.06; 4.44) | | | | 3.95 (3.27; 4.77) | | | 4.02 (3.35; 4.82) | | | 0.79 | | | | 5.04 (4.27; 5.94) | | | | | 5.03 (4.22; 5.99) | | | | | 4.86 (4.06; 5.82) | | | | 0.95 | | |
| *Pro- and Anti-Inflammatory* | |  | | | |  | | |  | | | |  | | | |  | | | | |  | | | | |  | | | | |
| IL-6 (pg/mL) | 1.86 (1.55; 2.24) | | | | 1.94 (1.61; 2.33) | | | 1.82 (1.52; 2.18) | | | 0.89 | | | | 2.37 (2.02; 2.79) | | | | | 2.32 (1.95; 2.76) | | | | | 2.33 (1.96; 2.76) | | | | 0.98 | | |
| IL-21 (pg/mL) | 1.24 (1.06; 1.44) | | | | 1.35 (1.12; 1.64) | | | 1.35 (1.15; 1.58) | | | 0.70 | | | | 1.41 (1.23; 1.63) | | | | | 1.41 (1.21; 1.65) | | | | | 1.60 (1.36; 1.88) | | | | 0.42 | | |
| GM-CSF (pg/mL) | 7.91 (6.78; 9.23) | | | | 7.38 (6.19; 8.80) | | | 6.83 (5.69; 8.19) | | | 0.48 | | | | 8.37 (7.17; 9.77) | | | | | 8.22 (7.03; 9.62) | | | | | 9.29 (8.00; 10.8) | | | | 0.51 | | |
| *Pro-to-Anti Inflammatory Ratios* | | |  | | | |  | | |  | | | |  | | | |  | | | | |  | | | | |  | | | |
| IL-6/IL-10 | 0.28 (0.22; 0.34) | | | | 0.27 (0.21; 0.34) | | | 0.32 (0.25: 0.40) | | | 0.50 | | | | 0.17 (0.13; 0.21) | | | | | 0.16 (0.13; 0.19) | | | | | 0.17 (0.13; 0.21) | | | | 0.92 | | |
| IL-1β/IL-10 | 0.21 (0.19; 0.23) | | | | 0.22 (0.20; 0.25) | | | 0.23 (0.20; 0.26) | | | 0.50 | | | | 0.20 (0.18;0.22) | | | | | 0.18 (0.17; 0.20) | | | | | 0.21 (0.19; 0.24) | | | | 0.14 | | |
| TNF-α/IL-10 | 0.36 (0.33; 0.39) | | | | 0.37 (0.34; 0.41) | | | 0.38 (0.35:0.42) | | | 0.70 | | | | 0.32 (0.29;0.35) | | | | | 0.33 (0.30; 0.36) | | | | | 0.36 (0.33; 0.39) | | | | 0.12 | | |
| CRP/IL-10 | 0.24 (0.18; 0.33) | | | | 0.21 (0.16; 0.29) | | | 0.24 (0.18; 0.32) | | | 0.80 | | | | 0.15 (0.12; 0.21) | | | | | 0.13 (0.11; 0.17) | | | | | 0.14 (0.10; 0.17) | | | | 0.67 | | |
| MIP-*1α*/IL-10 | 2.21 (1.95; 2.51) | | | | 2.19 (1.96; 2.46) | | | 2.21 (1.95; 2.50) | | | 1.00 | | | | 1.75 (1.56; 1.96) | | | | | 1.88 (1.68; 2.10) | | | | | 1.90 (1.70; 2.12) | | | | 0.54 | | |
| ITAC/IL-4 | 0.11 (0.09; 0.12) | | | | 0.11 (0.09; 0.13) | | | 0.11 (0.09; 0.13) | | | 0.92 | | | | 0.07 (0.07; 0.09) | | | | | 0.10 (0.08; 0.11) | | | | | 0.07 (0.07; 0.09) | | | | 0.024 | | |
| ITAC/IL- 5 | 5.52 (4.73; 6.43) | | | | 5.64 (4.71; 6.77) | | | 5.15 (4.42; 6.01) | | | 0.71 | | | | 3.41 (3.02; 3.85) | | | | | 3.83 (3.34; 4.41) | | | | | 3.59 (3.18; 4.04) | | | | 0.42 | | |
| ITAC/IL-10 | 1.11 (0.94; 1.29) | | | | 1.09 (0.92; 1.28) | | | 1.10 (0.95;1.29) | | | 0.99 | | | | 0.64 (0.57; 0.72) | | | | | 0.71 (0.62; 0.80) | | | | | 0.68 (0.61; 0.76) | | | | 0.52 | | |
| ITAC/IL-13 | 1.31 (1.08; 1.59) | | | | 1.24 (1.02; 1.51) | | | 1.17 (0.97; 1.41) | | | 0.71 | | | | 0.69 (0.58; 0.81) | | | | | 0.75 (0.62; 0.90) | | | | | 0.77 (0.63; 0.92) | | | | 0.67 | | |
