# Supplementary material for: Inflammation and salt in young adults: the African-PREDICT study
Source: Eur J Nutr. 2020 Jun 3;60(2):873–82. doi: 10.1007/s00394-020-02292-3 (PMC7900065; doi:10.1007/s00394-020-02292-3)
Supplement: Supplementary file 3 — Supplementary file3 (DOCX 22 kb) [file 394_2020_2292_MOESM3_ESM.docx]

**Inflammation and salt in young adults:**

**the African-PREDICT study**

**European Journal of Nutrition**

Simone H Crouch,^a^ Shani Botha-Le Roux,^a,b^ Christian Delles,^c^ Lesley A Graham,^c^ Aletta E Schutte^a,b,d^

*^a^ Hypertension in Africa Research Team (HART), North-West University, Potchefstroom, South Africa*

*^b^ MRC Research Unit: Hypertension and Cardiovascular Disease, North-West University, Potchefstroom, South Africa*

*^c^ Institute of Cardiovascular and Medical Sciences, College of Medical, Veterinary, and Life Sciences, University of Glasgow, Glasgow, United Kingdom*

*^d^ School of Public Health and Community Medicine, University of New South Wales; The George Institute for Global Health, Sydney, Australia*

Corresponding author: Prof. AE Schutte, School of Public Health and Community Medicine, Faculty of Medicine, Kensington Campus, High Street, Randwick, Sydney 2052, Email: a.schutte@unsw.edu.au

| **Table S3.** Partial correlations between Na^+^ and K^+^ and inflammatory mediators in total, black and white population. | | | | | | |  |
| --- | --- | --- | --- | --- | --- | --- | --- |
|  | **Total *** | | **Black** | | **White** | |  |
|  | Na^+^ (mmol/day) | K^+^ (mmol/day) | Na^+^ (mmol/day) | K^+^ (mmol/day) | Na^+^ (mmol/day) | K^+^ (mmol/day) |  |
| ***Pro-Inflammatory*** |  |  |  |  |  |  |  |
| CRP (mg/L) | - | r= -0.083 p= 0.010 | r= -0.22 p= 0.031 | - | - | r= -0.104 p= 0.018 |  |
| Fractalkine (pg/mL) | - | - | - | - | - | - |  |
| IFN-γ (pg/mL) | - | - | - | - | - | - |  |
| IL-1β (pg/mL) | - | - | - | - | - | - |  |
| IL-2 (pg/mL) | - | - | - | r= 0.129 p= 0.013 | - | - |  |
| IL-7 (pg/mL) | - | - | - | - | - | - |  |
| IL-8 (pg/mL) | - | - | - | - | - | - |  |
| IL-12 (pg/mL) | - | - | - | - | - | - |  |
| IL-17 A (pg/mL) | - | - | - | - | - | - |  |
| IL-23 (pg/mL) | - | - | - | - | - | - |  |
| ITAC (pg/mL) | - | - | - | - | - | - |  |
| MIP-1α (pg/mL) | - | - | - | - | - | - |  |
| MIP-1β (pg/mL) | - | - | - | - | - | - |  |
| MIP-3α (pg/mL) | - | - | - | - | - | - |  |
| TNF-α (pg/mL) | - | - | - | - | - | - |  |
| ***Anti-Inflammatory*** |  |  |  |  | - | - |  |
| IL-4 (pg/mL) | - | - | - | - | - | r= -0.092 p= 0.038 |  |
| IL-5 (pg/mL) | - | - | - | - | - | - |  |
| IL-10 (pg/mL) | - | - | - | - | - | - |  |
| IL-13 (pg/mL) | - | - | - | - | - | - |  |
| IL-6 (pg/mL) | - | - | - | - | - | - |  |
| IL-21 (pg/mL) | - | - | - | - | - | - |  |
| GM-CSF (pg/mL) | - | - | - | - | - | - |  |
| ***Pro-to-Anti Inflammatory Ratios*** | | | | | | |  |
| IL-6/IL-10 | - | - | - | - | - | - |  |
| IL-1β/IL-10 | - | - | - | - | - | - |  |
| TNF-α/IL-10 | - | - | - | - | - | - |  |
| CRP/IL-10 | - | r= -0.067 p= 0.041 | - | - | - | - |  |
| MIP-*1α*/IL-10 | - | - | - | - | - | - |  |
| ITAC/IL-4 | - | - | - | - | - | r= 0.115 p= 0.009 |  |
| ITAC/IL- 5 | - | - | - | - | - | - |  |
| ITAC/IL-10 | - | - | - | - | - | - |  |
| ITAC/IL-13 | - | - | - | - | - | - |  |
| Adjusted for: Age, sex and waist circumference. * Additionally adjusted for ethnicity. | | | | | | | |
