# Supplementary material for: Inflammation and salt in young adults: the African-PREDICT study
Source: Eur J Nutr. 2020 Jun 3;60(2):873–82. doi: 10.1007/s00394-020-02292-3 (PMC7900065; doi:10.1007/s00394-020-02292-3)
Supplement: Supplementary file 4 — Supplementary file4 (DOCX 214 kb) [file 394_2020_2292_MOESM4_ESM.docx]

**Inflammation and salt in young adults:**

**the African-PREDICT study**

**European Journal of Nutrition**

Simone H Crouch,^a^ Shani Botha-Le Roux,^a,b^ Christian Delles,^c^ Lesley A Graham,^c^ Aletta E Schutte^a,b^

*^a^ Hypertension in Africa Research Team (HART), North-West University, Potchefstroom, South Africa*

*^b^ MRC Research Unit: Hypertension and Cardiovascular Disease, North-West University, Potchefstroom, South Africa*

*^c^ Institute of Cardiovascular and Medical Sciences, College of Medical, Veterinary, and Life Sciences, University of Glasgow, Glasgow, United Kingdom*

Corresponding author: Prof. AE Schutte, Hypertension in Africa Research Team (HART), North-West University, Private Bag X6001, Potchefstroom, 2520, South Africa, Tel. +27 18 299 2444, Fax +27 18 285 2432, Email: [Alta.Schutte@nwu.ac.za](mailto:Alta.Schutte@nwu.ac.za)


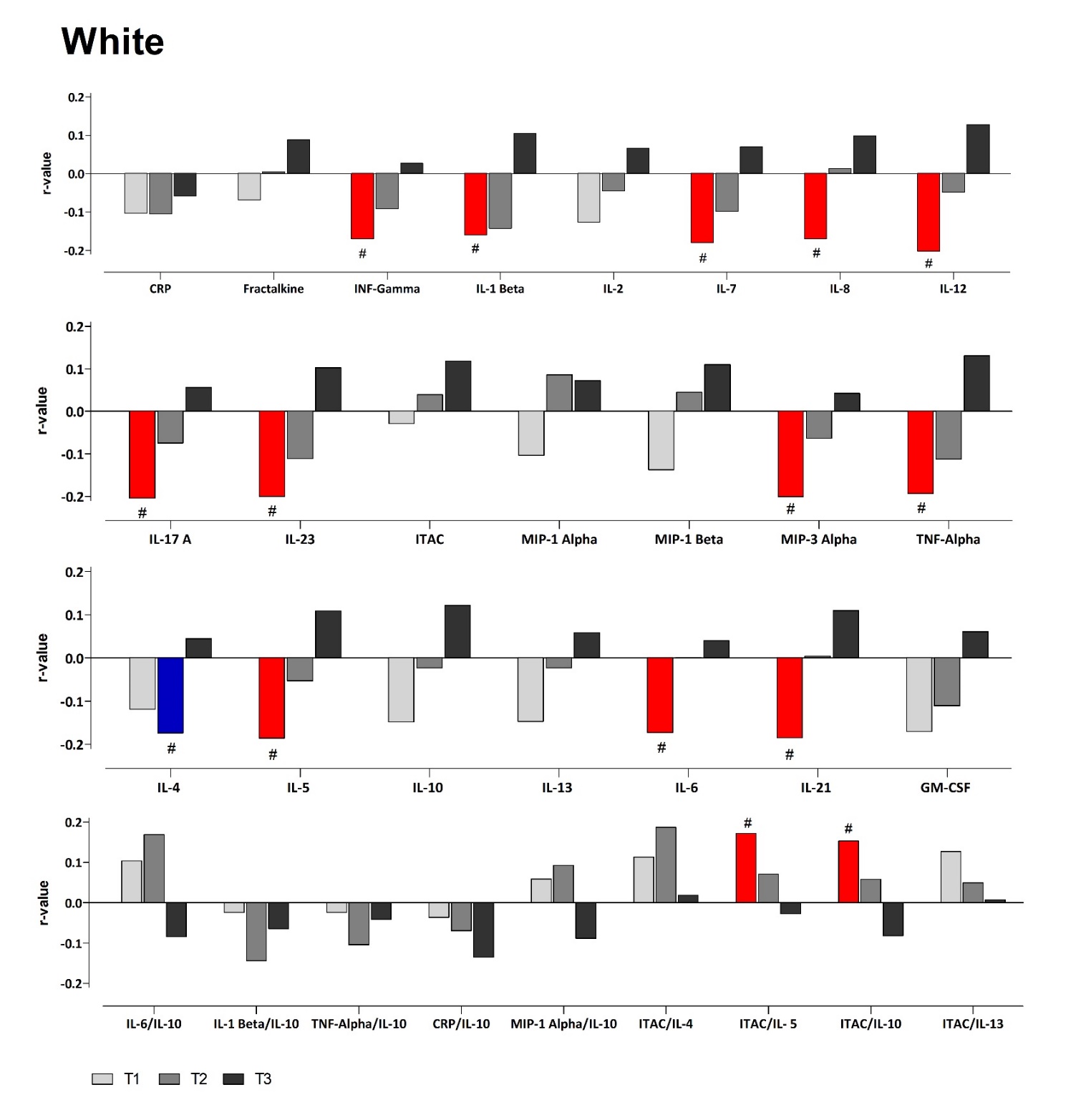


**Fig. S1** Partial correlations between 24 hr K^+^ and inflammatory mediators in white individuals, within 24h Na^+^ tertiles.

Each model was adjusted for: age, sex and waist circumference.

# indicates correlation with p<0.05.
